# Supplementary material for: Enhanced Tunnel Spin Injection into Graphene using Chemical Vapor Deposited Hexagonal Boron Nitride
Source: Sci Rep. 2014 Aug 26;4:6146. doi: 10.1038/srep06146 (PMC4143790; doi:10.1038/srep06146)
Supplement: Supplementary Information [file srep06146-s1.pdf]

Supplementary Information

Enhanced Tunnel Spin Injection into Graphene using Chemical Vapor Deposited Hexagonal Boron Nitride

M. Venkata Kamalakar, André Dankert, Johan Bergsten, Tommy Ive, Saroj P. Dash†*

Department of Microtechnology and Nanoscience, Chalmers University of Technology, SE-41296, Göteborg, Sweden

e-mail: *venkata.mutta@chalmers.se; †saroj.dash@chalmers.se

1. Fabrication details of Graphene/h-BN/Co spin transport devices

Graphene preparation

The graphene flakes were prepared on a clean SiO_2 (285 nm) highly doped silicon substrate with predefined Ti/Au markers. We used graphene exfoliated from highly oriented pyrolytic graphite (obtained from Advanced Ceramics) using the conventional scotch tape method. Graphene flakes were identified using a combination of optical and atomic-force microscopy. In our experiments, we used graphene flakes with widths of around 1.5 μm.

h-BN layer transfer

The atomically thin h-BN layer used as tunnel barriers in our experiment, was grown by chemical vapor deposition (CVD) on copper substrate and has high crystalline quality with ~95% coverage (obtained from Graphene supermarket). During the CVD process, h-BN grows on both sides of the copper foil (h-BN/Cu/h-BN). The h-BN on the top surface was first protected with thick PMMA (A11 e-beam resist), and the h-BN on the lower surface was removed by argon plasma etching. Then the PMMA/h-BN/Cu structure was kept in $FeCl_3$ solution for etching the Cu substrate. After complete removal of the Cu, the floating layer of PMMA/h-BN film was washed several times with deionized (DI) water. To remove possible chemical residues, the film was also treated with 10 % HCl for 20 min, followed by neutralization with DI water. The PMMA/h-BN film was then transferred onto the graphene flakes prepared on a SiO_2/Si chip (Fig. S1). The chip containing the PMMA/h-BN/graphene heterostructure was dried and baked at 150 °C for 5 minutes to improve the adhesion between the h-BN and the graphene. Finally, the PMMA layer was removed using hot (60 °C) acetone and the h-BN/graphene on the chip was annealed at 200 °C in a H_2/Ar atmosphere for 20 minutes. Careful transfer processes with thicker PMMA and the subsequent optimized drying and baking procedure ensured a uniform and ripple-free h-BN layer on the graphene. The h-

BN layer was further patterned to shapes covering the graphene flakes using electron-beam lithography and argon plasma etching (shown in Fig. S2). We patterned the h-BN layer (leaving it on the areas covering graphene regions) to avoid adhesion problems during bonding of the contact pad.

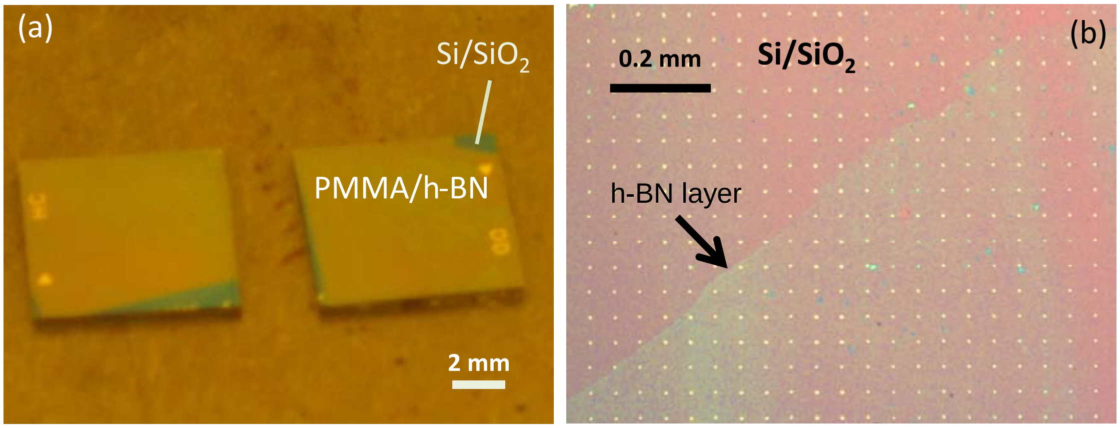

Fig. S1. (a) PMMA/h-BN transferred to 7 x 7 mm^2 SiO_2/Si chips. (b) Optical microscope image of the transferred CVD h-BN layer on Si/SiO_2 substrate.

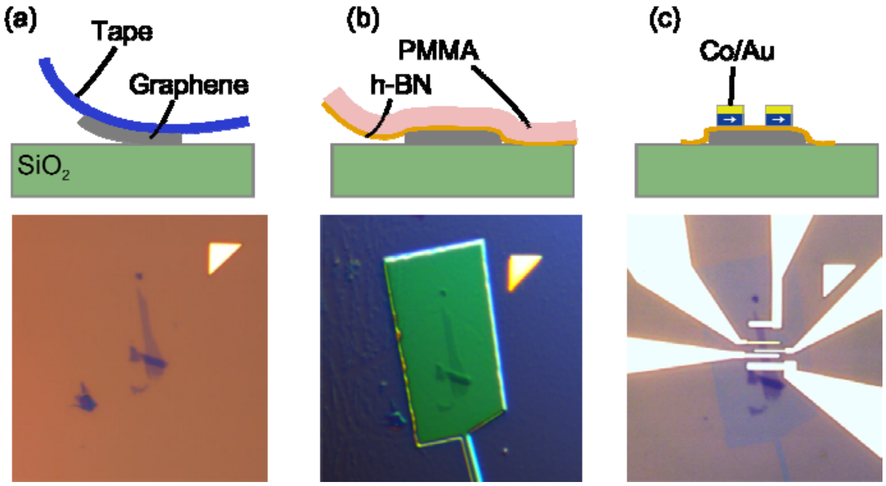

Fig. S2. (a) Graphene layers after exfoliation to a Si/SiO_2 substrate. (b) Resist covering regions of h-BN for patterning. (c) Final device showing graphene, patterned h-BN tunnel barrier along with the ferromagnetic tunnel contacts.

2. Characterization of h-BN tunnel barriers

The CVD grown hexagonal boron nitride obtained from graphene supermarket is characterized by the supplier as single layer hexagonal boron nitride from the Raman shift line (2568cm^{-1}). Also the datasheet provided by the suppliers involves scanning electron

microscopy (SEM) and Transmission electron microscopy (TEM) characterizations confirming the crystallinity of the layers. We have also performed AFM characterization to determine the thickness of the layers. The AFM studies of the CVD h-BN on Si/SiO_2, revealed a minimum layer thickness of ~ 5 Å (Fig. 1c in the main text) with mean thickness lying between 5 Å-10 Å (Fig. S3). We note that we also observed some regions (though much fewer) with higher thickness.

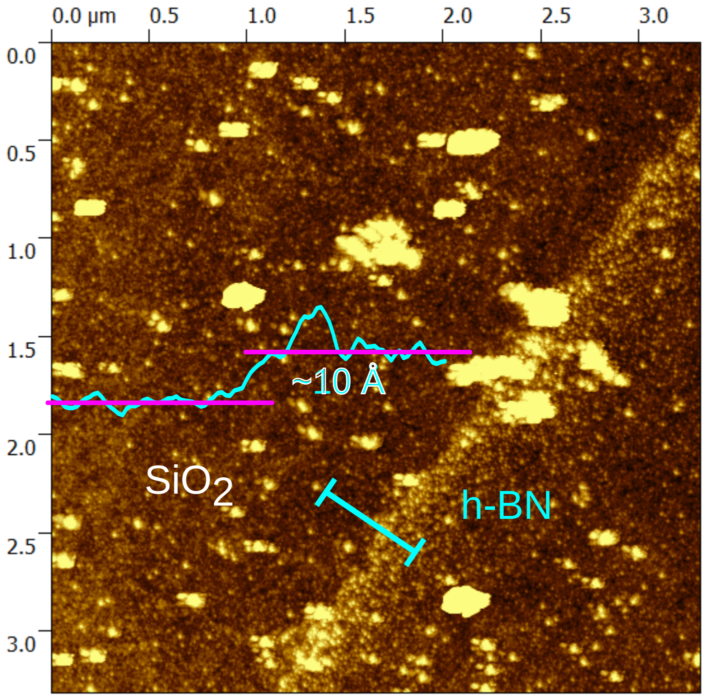

Fig. S3. AFM image showing thicker region of the h-BN (~10 Å) on Si/SiO_2 substrate.

Electrical measurements

Electrical characterization of the contacts and the device was performed using multi-terminal (such as 2 terminal (2T), 3 terminal (3T), 4 probe and 4 terminal non-local configurations) measurement configurations. The high contact resistance (when compared to transparent contacts), and its weak temperature dependence confirm the high quality of the h-BN tunnel barriers on graphene. Fig. S4 shows the tunnel characteristic curves for devices with h-BN in the order of increasing tunnel resistance. h-BN0 refers to devices without tunnel barrier. The IV curves show non-linear (Fig. S4a) at high bias voltages with linear behavior at low bias (Fig. S4b). In Fig. S5, we show the IV tunneling curves with weak temperature dependence for the spin injection contact of the device h-BN2.

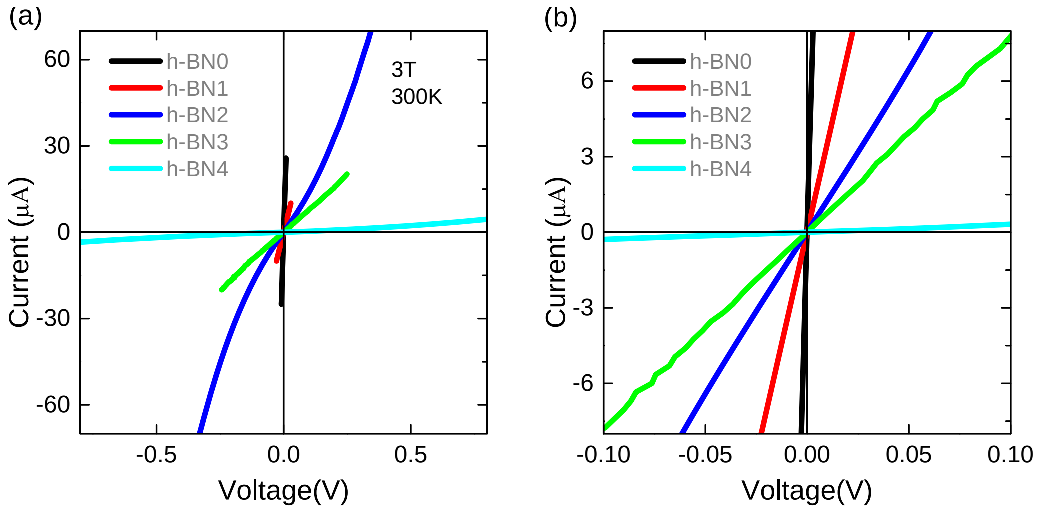

Fig. S4. (a) A comparison of three-terminal IV curves of injection h-BN tunnel contacts in different devices at 300 K. (b) Low bias region of the same plot.

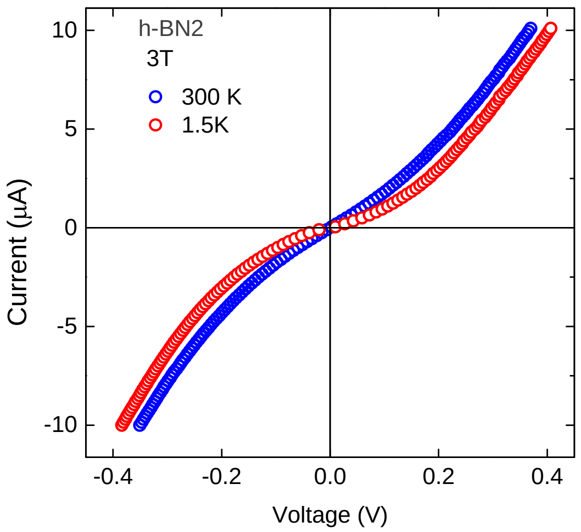

Fig. S5. Three terminal (3T) tunnelling IV characteristic curves of the device h-BN2 at 1.5 K and 300 K.

Tunnel barrier quality

The important criterion for tunnel barrier characterization is the weak temperature dependence of the tunnel resistance, which we have observed in our tunnel contacts. In addition, we have also been able to fit our IV curves, the Rowell fitting equation[1] for asymmetric barrier heights across the tunnel barrier junction, which is given by

$$G(V) = G_0\left[1-\left(\frac{4(2m)^{1/2}d}{3\hbar}\frac{\Delta\phi}{16\phi^{3/2}}\right)eV+\left(\frac{9}{128}\left(\frac{4(2m)^{1/2}d}{3\hbar}\right)^2\frac{1}{\phi}\right)(eV)^2\right],$$

where $G(V)$ is the differential conductance, G_0 is the zero bias conductance, m is the effective mass of electron, d, φ and $\Delta\varphi$ being the thickness, average barrier height and difference in barrier height across the junction respectively.

Scaling of h-BN tunnel resistance

The RA product as observed from previous reports[2] is also a criteria to resolve thin layers of h-BN. In Fig. S7, we highlight the RA of our devices in the scaling plot of RA vs. number of h-BN layers extracted from Britnell et.al[2]. In addition to the AFM data, from Fig.S7, we can infer that the majority of our devices showed resistances in the range corresponding to 1-2 layer thick h-BN.

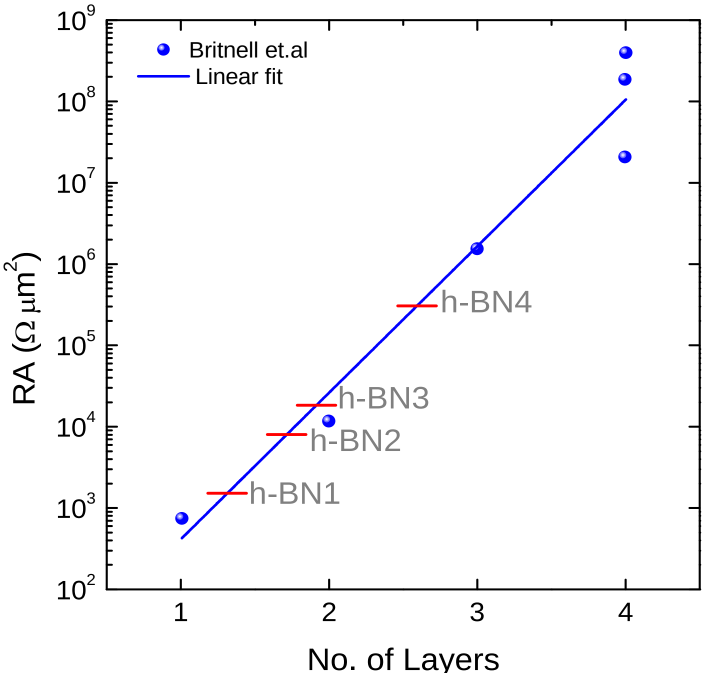

Fig. S6. Scaling of resistance area (RA) of h-BN as a function of the number of layers[2]. The red lines indicate the resistance levels of our devices.

3. Gate voltage dependence of graphene and tunnel barrier

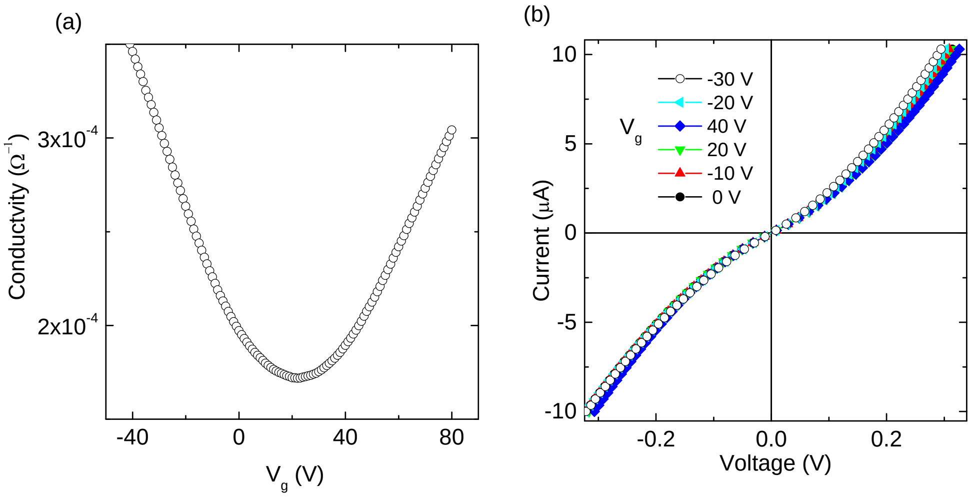

Fig. S7. (a) Dirac Curve of device h-BN3 (b) The variation of tunnel contact IV curves as a function of gate bias.

The channel resistance of the graphene is measured using the four-probe technique found to be 2 – 6 kΩ, showing regular gate-dependent Dirac curves, with mobility ~ 2000-3000 $cm^2 V^{-1} s^{-1}$ for 2-3 layer thick graphene employed in this study. A typical Dirac curve for device h-BN3 is shown in Fig. S7(a). Device showing similar behavior with Dirac point lying between 10-20 V and similar mobility were used for measurement of spin transport. In addition the tunnel barrier resistance of the contact did not seem to change with gate bias as shown in Fig. S7(b) . We note that all the spin transport measurement were performed at zero gate bias voltage.

References

1. Brinkman, W. F, Dynes, R.C., Rowell, J. M. Tunneling Conductance of Asymmetrical Barriers. *J. Appl. Phys.* **1970**, *41*, 1915.

2. Britnell, L.; Gorbachev, R. V; Jalil, R.; Belle, B. D.; Schedin, F.; Katsnelson, M. I.; Eaves, L.; Morozov, S. V; Mayorov, A. S.; Peres, N. M. R.; *et al.* Electron Tunneling through Ultrathin Boron Nitride Crystalline Barriers. *Nano Lett.* **2012**, *12*, 1707–1710.
